# 395. Early Predictors of Intensive Care Unit Admission among COVID-19 Patients in Qatar

**DOI:** 10.1093/ofid/ofab466.596

**Published:** 2021-12-04

**Authors:** Safae Abuyousef, Shaikha Alnaimi, Nabil E Omar, Reem Elajez, Eman Zeyad Ibrahim Elmekaty, Eiman Abdelfattah-Arafa, Raja Barazi, Rola Ghasoub, Ala Rahhal, Fatima Hamou, Maha K Al-Amri, Ahmed Karawia, Fatima Ajaj, Raja Alkhawaja, Ahmed Kardousha, Ahmed Awaisu, Adel Abou-Ali, Mohamad Khatib, Mohamed AbouKamar, Moza Al-Hail

**Affiliations:** 1 Department of Pharmacy, Heart Hospital, Hamad Medical Corporation, Doha, Qatar, Doha, Ad Dawhah, Qatar; 2 Department of Pharmacy, Hamad Bin Khalifa Medical City, Hamad Medical Corporation, Doha, Ad Dawhah, Qatar; 3 Pharmacy Department, National center for cancer care and research, Hamad Medical Corporation, Qatar, Doha, Ad Dawhah, Qatar; 4 Hamad Medical Corporation, Doha, Ad Dawhah, Qatar; 5 ncccr, doha, Al Khawr, Qatar; 6 Hamad medical corporation, Doha, Ad Dawhah, Qatar; 7 Home Health Care, Hamad Medical Corporation, Doha, Qatar, doha, Ad Dawhah, Qatar; 8 Department of Pharmacy, Hamad General Hospital, Hamad Medical Corporation, Doha, Qatar, Doha, Ad Dawhah, Qatar; 9 Hamad Medical corporation, Doha, Ad Dawhah, Qatar; 10 College of Pharmacy, Qatar University, Doha, Ad Dawhah, Qatar; 11 Astellas Pharma Global Development, Inc., Northbrook, Illinois; 12 Hamad General Hospital, Hamad Medical Corporation, Doha, Qatar, Doha, Ad Dawhah, Qatar; 13 Hamad Medical Corpoartion, Doha, Ad Dawhah, Qatar

## Abstract

**Background:**

Coronavirus disease (COVID-19) is associated with significant morbidity and mortality. This study aimed to explore the early predictors of intensive care unit (ICU) admission and in-hospital mortality among patients diagnosed with COVID-19.

**Methods:**

This was a case-control study of adult patients with confirmed COVID-19. Cases were defined as patients admitted to ICU during the period February 29 - May 29, 2020. For each case enrolled, one control was matched by age and gender.

**Results:**

A total of 1560 patients with confirmed COVID-19 were included. Each group included 780 patients with a predominant male gender (89.7%) and a median age of 49 years (interquartile range = 18). Predictors independently associated with ICU admission were cardiovascular disease (CVD) (adjusted odds ratio (aOR)=1.64, 95% confidence interval (CI): 1.16 - 2.32, p=0.005), diabetes (aOR=1.52, 95% CI: 1.08 - 2.13, p= 0.016), obesity (aOR=1.46, 95% CI: 1.03-2.08, p= 0.034), lymphopenia (aOR=2.69, 95% CI: 1.80-4.02, p< 0.001), high aspartate aminotransferase (AST) (aOR= 2.59, 95% CI: 1.53-4.36, p< 0.001), high ferritin (aOR=1.96, 95% CI: 1.40-2.74, p< 0.001), high C-reactive protein (CRP) (aOR=4.09, 95% CI: 2.81-5.96, p< 0.001), and dyspnea (aOR=2.50, 95% CI: 1.77-3.54, p< 0.001). Similarly, significant predictors of mortality included CVD (aOR=2.16, 95% CI: 1.32- 3.53, p=0.002), diabetes (aOR=1.77, 95% CI: 1.07-2.90, p=0.025), cancer (aOR=4.65, 95% CI: 1.50-14.42, p= 0.008), lymphopenia (aOR=2.34, 95% CI: 1.45-3.78, p= 0.001), and high AST (aOR= 1.89, 95% CI: 1.04-3.43, p=0.036).

Risk Factors for ICU admission among patients with COVID-19 (N=1560)

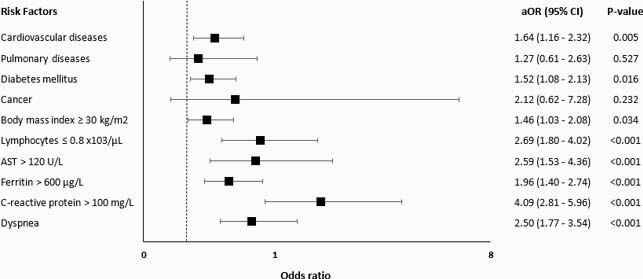

**Conclusion:**

Having CVD, diabetes, lymphopenia, and increased AST were independent predictors for both ICU admission and in-hospital mortality in patients with COVID-19. In addition, obesity, high ferritin, and CRP levels were associated with increased risk of ICU admission, while cancer was strongly associated with in-hospital mortality. Early identification and monitoring of patients at risk is essential in planning the level of care needed to prevent delay in medical intervention.

**Disclosures:**

**Adel Abou-Ali, PharmD, PhD**, **Astellas Pharma Global Development, Inc.** (Employee)

